# A Novel Mutation in PEX11β Gene

**DOI:** 10.22037/ijcn.v15i1.26129

**Published:** 2021

**Authors:** Hamid MALEKZADEH, Marjan SHAKIBA, Mehrdad YASAEI

**Affiliations:** 1 Department of pediatric endocrinology and metabolism, Mofid Children’s Hospital, Shahid Beheshti University of Medical Sciences, Tehran, Iran.

**Keywords:** PEX11β, peroxisomal disease, peroxisome biogenesis disorder, congenital cataract

## Abstract

PEX11β ([OMIM] 614920) mutation causes an extremely rare subgroup of peroxisomal biogenesis disorders, with only six cases reported to date. In this article, we reported a patient with episodic migraine-like attacks, delirium, mood and behavior change, polyneuropathy, and history of congenital cataract. Whole exome sequencing showed novel c.743_744delTCinsA mutation in the exon 4 of the PEX11β gene. In contrast to previously reported patients, our case presented milder features and extended the spectrum of the clinical phenotype of this mutation. This study helps to extend the phenotype of this syndrome; besides, recognizing novel mutation variants will provide a better genotype-phenotype correlation and improve clinical clues.

## Introduction

Peroxisomes are membrane-enclosed organelles involved in a variety of metabolic pathways including α- and β-oxidation of fatty acids and synthesis of bile acids, cholesterol, and plasmalogens (1). Mutations in genes encoding peroxisomal proteins are associated with two different groups of peroxisomal disorders: isolated enzyme deficiencies affecting specific metabolic pathways and peroxisome biogenesis disorders (PBDs) causing a defect in the assembly and maintenance of peroxisomes (2). PBDs, including Zellweger spectrum disorders and rhizomelic chondrodysplasia punctuate, are mainly caused by mutations in PEX proteins. These proteins, also known as peroxins, are encoded by 16 different PEX genes (3). The phenotypic presentation can range from mild to severe and early to late due to genetic heterogeneity (2).

PEX11 family members encode peroxisomal membrane proteins which coordinate peroxisome proliferation and maintenance and have a direct role in the metabolism of fatty acids (4, 5). Among PEX11 gene subgroups, recently, six patients have been reported to have PEX11β mutations ([OMIM] 614920) with various clinical presentations. In this article, we reported a patient with a novel mutation in the PEX11β gene and new clinical features.

## Case Report

A 15-year-old boy was referred to our hospital with presenting symptoms of nausea, vomiting, severe headache, delirium, and a complaint of mood and behavior change. He was the first child of consanguineous parents born at term with a birth weight of 3.1 kg. He had no history of intellectual disability, but a mild motor developmental delay was reported in his past medical history; he started walking at 18 months. He was diagnosed with bilateral cataracts at birth and underwent surgery in his 40 days of life, but no further workup was performed at that time. When he was 2.5 years old, he experienced three episodes of clonic seizure attacks during one month; however, EEG and MRI did not show abnormalities at that time. He did not experience any symptoms until 11 years of age when an episode of fever, confusion, and delirium occurred. CBC, blood biochemistry, lumbar puncture analysis, and brain MRI were all normal at that time, and a diagnoses of viral encephalitis was made. He experienced similar episodes at the age of 13. His parents stated that he exhibited behavioral and emotional problems like childish talk and mood instability during the attacks and turned normal after the episodes terminated. He also experienced headaches with migraine-like features during these episodes. The episodes resolved spontaneously within two weeks, and no specific trigger was noted.

Upon physical examination, he was disoriented to time and place. He exhibited delirious state and childish behaviors like engaging in baby talk and short attention spans. He had a mild dry skin, mostly visible on the extremities and his face. Moreover, he had an abnormal gait and neurologic examination detected normal muscle tone and strength but reduced symmetrical reflexes in lower extremities. No significant abnormalities were found in other examinations.

Initial blood tests of the patient were within normal limits, and his liver function tests, lipid profile, ammonia, and lactate showed no abnormality. Further, no pathologic results were found in primary metabolic screening for fatty acid oxidation disorders, organic acidemias, and amino acid disorders. Urine organic acids and homocysteine levels as well as plasma amino acids levels measured with high performance liquid chromatography were normal. Moreover, plasma levels of very-long-chain fatty acids (C26:0, C24:0/C22:0, and C26:0/C22:0) and pristanic and phytanic acids revealed no abnormalities. The auditory brainstem response test was performed, and no defects were reported. On the other hand, the nerve conduction velocity test revealed both sensory and motor polyneuropathy bilaterally in lower extremities.

With suspicion to mitochondrial or peroxisomal disorders, the mitochondrial treatment protocol (Vit B1, Vit B2, CoQ10, Vit E, b6, and L-carnitine selenium) was administered for the patient. The attack resolved in days with no significant clinical damage. 

Whole exome sequencing was requested due to the patient’s clinical features. DNA extracted from his blood was used to perform targeted gene capture using a custom capture kit. The obtained sequences were aligned to human reference genome (GRCh37/hg19) using the BWA program (6, 7) and analyzed using the Picard and GATK-Lite toolkit. The result indicated that the novel c.743_744delTCinsA mutation was located in the exon 4 of the PEX11B gene, leading to a frameshift and premature truncation of three amino acids downstream to the codon 248 (p.Leu248GInfsTer3). Sanger sequencing confirmed that the patient was homozygous for this variant. This novel variant was predicted to be damaging by Mutation Taster and considered as a pathogenic variant mutation of the disease.

## Discussion

PBDs are a group of genetic inborn errors of metabolism, in which defective biogenesis of peroxisomes leads to complex clinical and metabolic phenotypes. Peroxisome biogenesis is controlled by proteins that encompass processes involved in the control of peroxisome size and number, and other proteins intervene in the assembly of matrix and membrane proteins on peroxisomes (8). Proteins involved in these processes are peroxins encoded by PEX genes, and dysfunction in 14 identical peroxins are associated with human diseases. PEX11β which was first linked to human disease in 2012 plays a role in peroxisome division and membrane elongation (9-11).

In 2012, Ebberink et al. reported the first patient with a homozygous nonsense mutation in the PEX11β gene. The patient was a 26-year-old Dutchman of non-consanguineous parents, presenting with a history of congenital cataract, mild intellectual disability, bilateral progressive hearing loss, gastrointestinal problems, polyneuropathy, and recurrent migraine-like episodes (12). In 2016, Taylor et al. described five individuals with novel biallelic loss of function mutations in the PEX11β gene from three different families. They were presented with congenital cataract, intellectual disability, seizure, hearing loss, gastrointestinal complaints, behavioral problems, and dry skin. Moreover, skeletal involvements and short stature were observed in some cases (Table 1).

Episodic migraine-like attacks presented in our patient were similar to those in a case reported by Ebberink et al. (12). Although periodic mood change with childish behavior during attacks may be classified as a spectrum of behavioral disturbances observed in previous PEX11β mutations, it should be noted that the periodic and resolving aspects of this feature were unique in our case. Polyneuropathy, which was shown in the first reported case, was demonstrated with milder symptoms in our patient. Hearing loss, as well as abnormal mental development, gastrointestinal complaints, and skeletal disorders were not developed in our case. History of bilateral congenital cataract was present in all of the reported patients, suggesting PBDs to be considered among differential diagnoses for congenital cataract (13). Congenital cataract is inherited in both autosomal dominant and recessive fashions and affects 2.5 to 3.5 per 10000 births. Determining the genetic cause of congenital cataract could have significant clinical relevance by guiding genetic counseling and implementing early treatment strategies (14).

For many hereditary genetic disorders, disease severity is determined by the type of mutation and its impact on protein function. The milder phenotype in this patient suggests that the type and location of mutation in the PEX11β gene may be associated with the severity and divergence of symptoms. The novel mutation described in this article has not been reported previously. The consanguinity of parents is compatible with autosomal recessive inheritance described for this syndrome (13). Our findings expanded the spectrum and severity of the clinical phenotype associated with PEX11β variants. We also showed that PBDs could exist despite normal biochemical measurements, highlighting genetic analysis as an appropriate means for diagnosis of PEX11β variants when there is a high suspicion for presence of a systemic metabolic disease.

Because of the rarity of this disease, no treatment protocol has been established yet. Administering L-carnitine and the Co-Q10 enzyme in the first reported patient showed an overall improvement in his condition, although he still experiences migraine-like attacks (12). In our patient, a mitochondrial treatment protocol was initiated in the last attack; however, it was discontinued after the symptoms resolved.

**Table 1:** Summary of phenotypic features of the reported cases of PEX11β mutations

		Ebberink et al.	Taylor et al. (i)	Taylor et al. (ii)	Taylor et al. (iii)		Taylor et al. (iv)	Taylor et al. (v)		presented
Age / Sex		26 y / Male	23 y / Female	6 y / Male	6 y / Female		35 mo. / Male	23 mo. / Female		15 y / Male
Neurologic										
Muscular tone		Normal	Normal	Normal	Hypotonia		Hypotonia	Normal		Normal
Reflexes		Reduced	Reduced	Normal	Increased		Normal	Normal		Reduced
Hearing		Perceptive HL*	Normal	Sensorineural HL	Normal		Conductive HL	Normal		Normal
Other	Migraine-like episodes, Sensory disturbance, nystagmus		Nystagmus, Nonreactive pupils	Reduced muscular strength	Reduced muscular strength, Four-limb spasticity		Seizures	-	Seizures, Migraine-like episodes with confusion and delirium	
Eyes										
Bilateral congenital Cataract		+	+	+	+		+	+		+
Other		-	Divergent strabismus, Microphtalmia	Aphakic gkaucoma, Phtiasis	Aphakic gkaucoma		-	-		-
Development										
Intellectual disability		+	+	+	+		+	+		-
Sitting / walking		Normal / 1.5 y	Normal / Normal	1 y / 2 y	2 y / -		Normal / 16 mo.	Normal / Normal		Normal / 18 mo.
Speech		Normal	Normal	2 y	2 y		18 mo.	Normal		
Other		Regression following surgery	-	Regression following trauma, Behavioral & emotional problems	Behavioral problems		Delayed fine motor skills	Behavioral problems, Poor concentration		Behavioral & emotional problems
Other systems		Dry, scaly skin	Short stature, flat feet with hallux valgus, dry skin, GI^†^ problems	Short stature, Flat feet with hallux valgus, Dry skin, GI problems		FTT, Scoliosis, Dry skin	FTT, High forehead	-		Dry skin


*Hearing Loss, ^†^ Gastrointestinal

## Conclusion

PEX11β mutation causes an extremely rare subgroup of peroxisomal biogenesis disorders, with only seven cases reported to date. The main feature of the disease is congenital cataract that was developed in all of the reported cases. Normal metabolic tests in our case drew attention to the utility of genetic analysis when presence of a systemic metabolic disorder is likely. Extending the phenotype of this syndrome in addition to recognizing novel mutation variants will provide a better genotype-phenotype correlation and enhance clinical diagnostic clues.
